# The prevalence change of hyperlipidemia and hyperglycemia and the effectiveness of yearly physical examinations: an eight-year study in Southwest China

**DOI:** 10.1186/s12944-018-0724-6

**Published:** 2018-04-04

**Authors:** Wei Gan, Ying Liu, Kai-Hong Luo, Shan-Shan Liang, Hui Wang, Meng Li, Yan-Xing Zhang, Heng-Jian Huang

**Affiliations:** Department of Laboratory Medicine, West China Hospital, Sichuan University, No. 37 Guoxue Alley, Wuhou district Chengdu, Sichuan, 610041 China

**Keywords:** Annual physical examination, Serum lipids, Serum glucose, Hypercholesterolemia, Hyperglycemia

## Abstract

**Background:**

The aim of this study was to investigate the prevalence changes of hyperlipidemia and hyperglycemia from 2009 to 2016 and the effectiveness of yearly physical examinations to hyperlipidemia and hyperglycemia prevention in Chengdu.

**Methods:**

A total of 794 residents (499 males) who have undergone annual health check-ups for 8 consecutive years (from 2009 to 2016) in Chengdu, a city in southwest China were selected as the follow-up group, 7226 residents in 2009 and 75,068 residents in 2016 who underwent health examinations in the same hospital were chosen to be the contemporary control group. The concentration of fasting serum triglyceride(TG), total cholesterol(TC), low density lipoprotein cholesterol(LDL-C), high density lipoprotein cholesterol (HDL-C) and glucose were measured and compared among these groups.

**Results:**

There was a clear rise in the prevalence of hypercholesterolemia and hyperglycemia from 2009 to 2016 (*p* < 0.05). The follow-up group didn’t show difference in levels of serum lipids and glucose compared with the general population after an 8-years’ consecutive physical examination (*p* > 0.05), the follow-up cohort in the 8th year exhibited significant increases in serum total cholesterol and glucose compared with the 1st year (*p* < 0.05).

**Conclusion:**

The prevalence of hypercholesterolemia and hyperglycemia were increased significantly from 2009 to 2016. Annual physical examination didn’t show a positive effect in the prevention of hypercholesterolemia and hyperglycemia. Health education should be improved to ensure the fulfillment of the preventive objective of yearly physical examination.

## Background

Coronary heart disease (CHD) is the leading cause of death both in worldwide and China [1, 2]. In 2015, cardiovascular diseases (CVD) became the first leading cause of death for Chinese, 45.01% deaths in rural area and 42.61% deaths in urban area were caused by CVD [3].

Hyperlipidemia, especially elevated levels of low-density lipoprotein (LDL) cholesterol, is an independent risk factor for cardiovascular diseases [4]. The China Health and Nutrition Survey in 2012 [5] revealed that the prevalence of total cholesterol (TC) ≥ 6.22 mmol/L among males and females aged over 18 were 4.7 and 5.1%, respectively, and the prevalence rates of triglyceride (TG) ≥ 2.26 mmol/L among males and females were 16.7 and 9.8%, respectively.

Diabetes is also a major risk factor for cardiovascular disease, the prevalence of diabetes is increasing constantly in China. The overall prevalence of diabetes was estimated to be 11.6% in the Chinese adult population, with 12.1% among men and 11.0% among women [6].

Early detection of dyslipidemia and hyperglycemia is an important strategy for early prevention and treatment. Nowadays, yearly health examination, including serum lipid and glucose screening has become a welfare provided by many employers. Despite no evidence in favour, such kind of periodic physical examination is considered an important and effective measure in the prevention of illness, and is very popular national-wide.

This study aimed to answer the scientific question “How much do yearly health examinations do to the control of hyperlipidemia and hyperglycemia”. Moreover, this study also investigated the changes in the prevalence and incidence rates of hyperlipidemia and hyperglycemia from 2009 to 2016; and explored the relationship between age/sex and serum lipids and glucose.

## Methods

### Participants

All the participants were recruited from the physical examination center, West China Hospital, Sichuan University. A total of 794 local residents who started with a yearly health examination in 2009 for 8 consecutive years (from 2009 to 2016) were selected as the follow-up group, which was used to investigate the effectiveness of yearly physical examination to the control of hyperlipidemia and hyperglycemia. All the residents who went for health checkups in 2009 and 2016 were selected to be the contemporary control group and used to investigate the prevalence changes of hyperlipidemia and hyperglycemia from 2009 to 2016. All the participants were subdivided into 10-year age classes (≤30 years, 31–40 years, 41–50 years, 51–60 years, 61–70 years, ≥71 years). All the participants were from urban area of Chengdu city, Sichuan province of China. They were all common staffs of local company or agency, no special records about their life style were found in the subjects.

### Serum lipids and glucose measurement

Serum TG, TC, LDL-C, HDL-C and serum glucose levels were measured after at least a 10-h overnight fasting among all study participants, the measurements were performed on a Cobas8000 system (Roche Diagnostics GmbH, Germany) in the Department of Laboratory Medicine, West China Hospital, Sichuan University, which is a College of American Pathologists (CAP)-accredited laboratory.

### Diagnostic criteria

According to the guidelines on prevention and treatment of blood lipid abnormality in Chinese adults [7], TC ≥ 6.2 mmol/L was defined as hypercholesterolemia and TG ≥ 2.3 mmol/L was diagnosed as hypertriglyceridemia. T2DM was identified according to the criteria of Chinese Diabetes Society [8], fasting serum glucose ≥7.0 mmol/L was defined as hyperglycemia.

### Strategies for evaluating the effectiveness of yearly physical examination to hyperlipidemia and hyperglycemia control

Three strategies were used to evaluate the effectiveness of consecutive physical examination. 1) A cross-sectional comparison: Compare the levels of TG, TC and glucose between the 8th year of follow-up group and 2016 of control group. 2) A longitudinal comparison: Compare the levels of TG, TC and glucose between the 1st and the 8th physical examination year in the follow-up group. 3) Calculate the numbers of participants who had a bad hyperlipidemia or hyperglycemia control during the 8 years in the follow-up group. A “bad hyperlipidemia or hyperglycemia control” is defined as persons with fasting serum triglyceride ≥2.3 mmol/L, or cholesterol ≥6.2 mmol/L, or blood glucose ≥7.0 mmol/L for ≥3 times among the total 8 examinations.

### Statistical analysis

All statistical analyses were performed using the SPSS 16.0 software program (SPSS Inc. USA). Serum lipids and glucose concentration distributed normally and were expressed as the mean ± standard deviation. Differences between gender groups were assessed by independent t-test. Differences among age groups were tested by one-way ANOVA, followed by S-N-K test for equal variances assumed and Dunnett’s T3 test for equal variances not assumed. All *p* values were two-sided, and a *p* value < 0.05 was considered statistically significant.

## Results

### The relationship between age/sex and serum lipids and glucose

The number of the participants in each age group were listed in Table 1, the year of age spanned from 12 to 98. Data in 2016 were used to investigate the relationship between age/sex and these measured variables.

**Table 1:** Numbers of participants in each age group

	2009		Total	2016		Total	1st year of annual checkup		Total	8th year of annual checkup		Total
Age group	Male	Female		Male	Female		Male	Female		Male	Female	
≤30y	196	185		7268	8136		42	33		3	5	
31-40y	860	769		10,976	8986		111	80		60	47	
41-50y	1185	1016		10,557	8478		153	99		137	109	
51-60y	778	798		6772	4635		126	50		156	85	
61-70y	362	417		2609	2075		47	26		80	43	
≥71y	316	344		2699	1877		20	7		50	19	
Total	3697	3529	7226	40,881	34,187	75,068	499	295	794	486	308	794

The change patterns of serum lipids and glucose with aging were different from each other (Table 2). In detail, TG in female exhibited the most obvious age-dependent increase, the level of TG in ≤30 year group was 0.88 ± 0.44 mmol/L, a 1.76-fold increase was found in ≥71 year group (1.55 ± 0.73 mmol/L) (*p* < 0.001). Glucose in both gender also exhibited a demonstrated age-dependent increase, a 1.25- and 1.26-fold higher level for male and female were present in ≥71 year group compared with the ≤30 year group (both *p* < 0.001), respectively. HDL-C rose slightly during all the life-span, for male, a 1.12-fold increase were found in the ≥71 year group compared with the ≤30 year group (*p* < 0.001), for female, even more slightly changes were present from the young to the old. TG in male, TC and LDL-C in both gender followed the pattern of “first increase, and then decrease slightly”. However, the levels of the three variables in ≥71 year group were still higher than ≤30 year group. The turning points from “increase to decrease” were at the age of 50, 60 and 60 for TG in male, TC and LDL-C in both genders, respectively.

**Table 2:** The relationship between age/sex and serum lipids and glucose

	TG		TC		LDL-C		HDL-C		Glucose	
Age group	Male	Female	Male	Female	Male	Female	Male	Female	Male	Female
≤30y	1.42 ± 0.84	0.88 ± 0.44^#^	4.58 ± 0.83	4.38 ± 0.73^#^	2.57 ± 0.67	2.20 ± 0.57^#^	1.38 ± 0.32	1.78 ± 0.38^#^	4.96 ± 0.63	4.86 ± 0.44^#^
31-40y	1.69 ± 0.94^*^	1.00 ± 0.52^*,#^	4.83 ± 0.84^*^	4.54 ± 0.78^*,#^	2.74 ± 0.67^*^	2.35 ± 0.62^*,#^	1.34 ± 0.33^*^	1.74 ± 0.39^*,#^	5.14 ± 0.83^*^	5.00 ± 0.55^*,#^
41-50y	1.85 ± 0.99^*^	1.17 ± 0.62^*,#^	5.04 ± 0.88^*^	4.90 ± 0.83^*,#^	2.87 ± 0.72^*^	2.62 ± 0.67^*,#^	1.36 ± 0.34^*^	1.76 ± 0.41^*,#^	5.42 ± 1.26^*^	5.14 ± 0.66^*,#^
51-60y	1.78 ± 0.94^*^	1.45 ± 0.82^*,#^	5.05 ± 0.91^*^	5.40 ± 0.91^*,#^	2.88 ± 0.73^*^	2.98 ± 0.74^*,#^	1.38 ± 0.36	1.77 ± 0.44^#^	5.79 ± 1.68^*^	5.42 ± 1.00^*,#^
61-70y	1.55 ± 0.78^*^	1.56 ± 0.78^*^	4.99 ± 0.95^*^	5.37 ± 0.95^*,#^	2.82 ± 0.76^*^	2.95 ± 0.77^*,#^	1.46 ± 0.38^*^	1.74 ± 0.45^*,#^	6.06 ± 1.73^*^	5.82 ± 1.44^*,#^
≥70y	1.38 ± 0.67^*^	1.55 ± 0.74^*,#^	4.87 ± 0.96^*^	5.30 ± 1.06^*,#^	2.69 ± 0.77^*^	2.84 ± 0.85^*,#^	1.54 ± 0.44^*^	1.79 ± 0.49^#^	6.20 ± 1.68^*^	6.11 ± 1.63^*,#^

^*^: *p* < 0.001 compared with sex-matched ≤30 year group

^#^: *p* < 0.001 compared with age-matched male

Under the age of menopause, males always exhibited higher levels of serum lipids and glucose compared with age-matched female (*p* < 0.001) except for HDL-C. However, females experienced their highest increase during the menopausal period, and decreased much more slightly than male over the age of the “turning point”. As a result, female showed higher levels of serum lipids and glucose than male in the latter age stages.

### The changes in the prevalence of hyperlipidemia and hyperglycemia from 2009 to 2016

According to the diagnostic criteria, The incidence rates of hypertriglyceridemia, hypercholesterolemia and hyperglycemia in 2009 and 2016 were listed in Table 3. Since the number of participants in ≤30 year group and ≥ 71 year group in 2009 were small (see Table 1), to avoid introducing bias in calculating the incidence rate, we merged ≤30 year group and 31–40 year group to be ≤40 year group, 61–70 year group and ≥ 71 year group to be ≥61 year group.

**Table 3:** Percentages of hypertriglyceridemia, hypercholesterolemia and hyperglycemia in 2009 and 2016

	% with TG ≥ 2.3 mmol/L				% with TC ≥ 6.2 mmol/L				% with Glucose ≥7.0 mmol/L			
Age group	Male		Female		Male		Female		Male		Female	
	2009	2016	2009	2016	2009	2016	2009	2016	2009	2016	2009	2016
≤40y	26.5	17.0	1.3	2.1	3.8	5.3	1.0	2.2	2.1	1.1	0.0	0.2
41–50y	30.1	24.9	7.0	5.4	4.2	9.5	1.9	6.7	4.7	5.3	1.1	1.3
51–60y	23.8	22.5	13.4	11.5	4.2	10.4	9.8	17.9	7.3	11.0	4.3	4.0
≥61y	15.0	11.9	20.6	13.2	6.3	8.9	15.2	18.1	10.2	17.5	10.1	12.8

Concerning the year of 2016, the highest incidence rate of hypertriglyceridemia in male was 24.9% in 41–50 year group, 13.2% in ≥61 year female group. The maximum incidence rate of hypercholesterolemia in male was 10.4% in 51–60 year group, 18.1% in ≥61 female year group. As for hyperglycemia, participants in ≥61 year group exhibited the highest prevalence rate, which was 17.5 and 12.8% in male and female, respectively.

The year of 2016 witnessed great changes in the lipids profile and glucose in the past 8 years. The incidence rate of hypertriglyceridemia decreased in all the age groups except for the ≤40y female group. A significant increase in hypercholesterolemia prevalence was seen in all the age groups, the highest increase rate was 3.5 folds in 41–50 year group in female. The incidence rate of hyperglycemia also showed an obvious increase from 2009 to 2016 in all the age group except for ≤40 year in male. A 1.7-fold increase was seen in ≥61 year male group.

### Effectiveness of yearly physical examination to hyperlipidemia and hyperglycemia control

By using the 3 listed strategies, we got the following results (Table 4). There were no significant differences in TG, TC and glucose between the 2016 control group and the 8th year follow-up group (all *p* > 0.05). For the follow-up group, TC and glucose increased significantly from the 1st to the 8th year, while TG showed a slightly decrease (Table 4). Of the total 794 participants, 160 had a bad hypertriglyceridemia control, 36 had a bad hypercholesterolemia control, and 68 had a bad hyperglycemia control.

**Table 4:** Levels of TG, TC and glucose in 2016 of the control group, the 1st and 8th physical examination year of the follow-up group

		≤40y		41–50y		51–60y		≥61y	
		Male	Female	Male	Female	Male	Female	Male	Female
TG	2016	1.59 ± 0.94	0.88 ± 0.43	1.85 ± 0.99	1.17 ± 0.62	1.78 ± 0.94	1.45 ± 0.75	1.46 ± 0.73	1.55 ± 0.76
	1st year	1.81 ± 1.25	0.95 ± 0.48	2.15 ± 1.44	1.10 ± 0.61	1.87 ± 0.99	1.45 ± 0.89	1.74 ± 0.83	1.87 ± 1.15
	8th year	1.73 ± 1.15	0.98 ± 0.45	1.75 ± 0.91^*^	1.10 ± 0.55	1.70 ± 0.84	1.75 ± 0.78	1.53 ± 0.79^*^	1.65 ± 0.83
TC	2016	4.73 ± 0.84	4.37 ± 0.72	5.04 ± 0.88	4.90 ± 0.83	5.05 ± 0.91	5.40 ± 0.91	4.93 ± 0.95	5.33 ± 1.00
	1st year	4.34 ± 0.72	4.20 ± 0.89	4.81 ± 0.87	4.39 ± 0.67	4.69 ± 0.83	4.91 ± 0.85	4.84 ± 0.72	5.23 ± 0.93
	8th year	4.77 ± 0.78^*^	4.61 ± 0.75^*^	4.94 ± 0.87	4.90 ± 0.80^*^	5.05 ± 0.87^*^	5.22 ± 1.02^*^	4.95 ± 0.93	5.29 ± 1.01
Glucose	2016	5.06 ± 0.76	4.86 ± 0.44	5.42 ± 1.26	5.14 ± 0.66	5.79 ± 1.68	5.42 ± 1.00	6.13 ± 1.71	5.96 ± 1.54
	1st year	4.53 ± 0.41	4.49 ± 0.38	4.83 ± 0.86	4.65 ± 0.46	4.91 ± 1.00	4.89 ± 0.75	5.00 ± 0.84	4.96 ± 0.78
	8th year	5.14 ± 0.45^*^	5.03 ± 0.45^*^	5.33 ± 0.78^*^	5.10 ± 0.51^*^	5.55 ± 1.31^*^	5.38 ± 0.77^*^	5.88 ± 1.44^*^	5.69 ± 0.93^*^

^*^: *p* < 0.05 compared with age-matched group of the 1st physical examination year

## Discussion

Periodic physical examination is indeed very important in some situations, such as cancer screening [9]. However, its utility in the control of hyperlipidemia and hyperglycemia has not been fully elucidated. In this study, we firstly investigated the relationship between age/sex and serum lipids and glucose, it was demonstrated that TG in male followed the pattern of first increase, then decrease. TG in female showed an obvious increase with aging. TC and LDL-C in both genders followed the pattern of increase, decrease. HDL-C and glucose in both genders followed an age-dependent increasing manner. Which is similar to the report of Loh [10] and Harman [11]. These different lipids and glucose profiles in different age/sex persons have shown a need for age/sex dependent hyperlipidemia and hyperglycemia diagnostic cutoff value. Moreover, a clear “menopause phenomenon” which referred to that females showed a clear rise in the levels of lipids and glucose than males during the menopausal period was seen in all the groups, this phenomenon might be attributed to estrogen withdrawal in the menopausal period and indicated the need for special attention for menopausal women.

The levels of total cholesterol, LDL-C and glucose increased significantly during the 8 years, which is in accordance with the report [5, 6, 12–15], and partially in agreement with Kheirandish M [16] who showed a favorable trends in TC, triglycerides, HDL-C, but increased trends in glucose level in a 10 years follow-up. Our results showed an urgent need for population-based education on health eating and physical activity. In contrast with the reported data, TG decreased from 2009 to 2016 in our study. Firstly, we rechecked the history records of “proficiency test” which was organized by College of American Pathologists (CAP) and didn’t find any bad history records. After excluding the measurement error, we noticed that the physical examination center has delivered an extensive pre-examination education to the participants through various ways in recent years, therefore, we concluded that participants were in a more strictly fasting state in 2016 compared with 2009, which could give a lower TG results.

We evaluated the effectiveness of yearly physical examination to hyperlipidemia and hyperglycemia control through 3 aspects (see Methods), the results showed that after 8 years’ consecutive physical examination, the cohort were not “healthier” than the general population. Compared with the 1st year, the cohort in the 8th year showed significant increases in serum total cholesterol and glucose. Moreover, some participants showed a bad hyperlipidemia and hyperglycemia control. Based on these results, we demonstrated that effectiveness of periodic physical examination to the control of hyperlipidemia and hyperglycemia was limited. Very few researches focused on the same topic as we did. Lau et al. [17] showed a large population-based multifactorial screening on 10-year incidence of diabetes did not have a preventive effect on diabetes at population level.

As it is known, early stage of hyperlipidemia and hyperglycemia do not cause any discomfort to the body, but once be diagnosed, one needs to start treatment of therapeutic lifestyle change (TLC), including decline in energy intake and increase in physical activity. Lack of consistent health education and general practitioner in China makes these sub-healthy people difficult to insist on TLC treatment. The awareness, treatment, and control rate of dyslipidemia were low among adults in China [18]. Several researches have emphasized the importance of good lifestyle. CHOI et al. [19] have shown that the intervention group (who had consistent diet consultation) had a much better serum lipids control than the control group. Lu et al. [20] found that nursing interventions in public health centers demonstrated the effect of significantly reducing the values of blood pressures and blood sugars of the elderly in communities. However, a lack of lifestyle recommendations and use of preventive medication is a common problem. A study in Australia found that the vast majority of individuals at risk of or with CVD did not achieve preventive recommendations [21].

The limitation of this study is that the medication information of the participants were not included, which may influence the results of the relationship between serum lipids and glucose and age/sex (Table 2).

## Conclusions

Increasing age and estrogen withdrawal in perimenopause are dangerous factors for hyperlipidemia and hyperglycemia. The prevalence of hypercholesterolemia and hyperglycemia were increased significantly from 2009 to 2016. Annual physical examination didn’t show a positive effect in the prevention of hypercholesterolemia and hyperglycemia. We have well-established systems to find out the health problems, but more attention should be paid on the improvement of health education and popularization of general practitioners to ensure the fulfillment of the preventive objective of yearly physical examination.

## Abbreviations

Glu: Glucose
HDL-C: High density lipoprotein-cholesterol
LDL-C: Low density lipoprotein –cholesterol
TC: Total cholesterol
TG: Triglyceride
